# First reported *TRIB1* copy number loss in myelodysplastic syndrome (MDS) revealed by single nucleotide polymorphism array (SNP-array) with patient-matched control

**DOI:** 10.1016/j.lrr.2025.100542

**Published:** 2025-09-01

**Authors:** Kun Chi, Lili Song

**Affiliations:** a Department of Clinical Laboratory, Qingdao Women and Children’s Hospital, Women and Children’s Hospital, Qingdao University, 266034, Qingdao, China; b Department of Clinical Laboratory, Qingdao Public Health Clinical Center, 266034, Qingdao, China

**Keywords:** Copy number variations, Uniparental disomy, SNP-array analysis, *TRIB1*

## Abstract

A 30-year-old woman with MDS with low blasts (MDS-LB) presented a somatic 67-kb copy number loss at 8q24.13 involving *TRIB1*, detected by single nucleotide polymorphism (SNP) array. Bone marrow analysis showed no mutations in common MDS genes (*TP53, ASXL1, TET2, RUNX1*) or karyotypic abnormalities (46, XX). Using oral epithelial DNA as a patient-matched control, SNP-array identified four hereditary uniparental disomies (UPDs) and a somatic *TRIB1*-containing deletion at 8q24.13. This deletion likely caused *TRIB1* haploinsufficiency, reducing control over dysplastic clones and driving progression to MDS with increased blasts 2 (MDS-IB2) over three years. This first report of *TRIB1* copy number loss in myeloid disorders highlights the value of SNP-array with patient-matched controls in distinguishing somatic variants, expanding MDS’s genetic profile and underscoring *TRIB1*’s context-dependent roles in oncogenesis.

## 1 Introduction

Myelodysplastic syndrome (MDS) is a group of acquired clonal disorders of hematopoietic stem cells, characterized by severe dysregulation of hematopoietic function. These disorders are distinguished by abnormal development and ineffective hematopoiesis of one or more myeloid cell lines, with a risk of transformation into acute leukemia. The heterogeneity of clinical manifestations in MDS, the diversity of bone marrow cell morphology, and genetic heterogeneity make its diagnosis and prognosis challenging. For the diagnosis of MDS, a comprehensive analysis of various clinical indicators and cytological indicators is essential. Simultaneously, chromosomal aberrations play a crucial role in the prognosis in MDS.

With the advent of high-throughput single nucleotide polymorphism array (SNP-array), many subtle chromosomal variations, such as copy number variations (CNV) and uniparental disomy (UPD), have been identified in patients with MDS. This has led to the discovery of many genes associated with MDS pathogenesis, significantly enhancing understanding of the cytogenetic and molecular genetic mechanisms of MDS disease progression. However, the presence of non-pathogenic genetic variations can lead to false-positive results in SNP-array testing, potentially misleading diagnostics and treatment for patients. Therefore, eliminating the interference of these genetic variations has important clinical significance. Accordingly, we employed the SNP-array to conduct a comparative analysis of chromosomal variations from a patient whose molecular mechanisms were unknown at diagnosis.

## 2 Case presentation

The patient is a 30-year-old female with symptoms of dizziness and fever, who denied previous medical history or family history. Her hemoglobin was measured at 90 g/L, platelet count at 75 × 10^9/L, and white blood cell count at 3.0 × 10^9/L (absolute neutrophil count at 1.2 × 10^9/L) with no blast cells observed in the peripheral blood. Bone marrow examination showed hyperactive trilineage hematopoiesis with dysplastic changes visible, and a myeloid to erythroid ratio of 0.48:1, with a blast cell proportion of 4.5 %. She was diagnosed with MDS with low blasts (MDS-LB) according to the 2022 WHO classification of MDS [1].

**Fig. 1:**
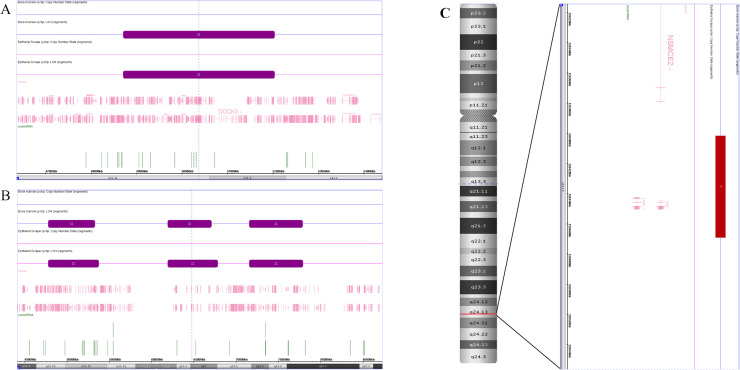
**A**. Lesion on chromosome 3, the purple bars represent the UPD regions, which are visible in both the bone marrow sample (above, purple) and the oral epithelial cells sample (below, pink), with largely consistent sizes and locations. **B**. Lesions on chromosome X, the three purple bars represent three UPD regions, which are visible in both the bone marrow sample (above, purple) and the oral epithelial cells sample (below, pink), with their sizes and locations also being largely consistent. **C**. Lesions on chromosome 8, the red bar represents the missing region that exists only in the bone marrow sample (above, purple) and is not seen in the oral epithelial cells sample (below, pink), covering the candidate pathogenic gene *TRIB1* in the missing area.

The G-banding result revealed a karyotype of 46, XX, with no abnormal chromosomes detected. Additionally, no mutations were detected in *TP53, ASXL1, TET2* or *RUNX1.* According to the CytoScan™ HD Array instructions and our criteria to determine disease-specific aberrations [2], we detected five significant chromosomal variations in the patient's bone marrow samples. Among these, four UPDs were identified: one on the short arm of chromosome 3 (3.3 Mb) and three on the X chromosome (5.5 Mb, 5.2 Mb, and 6.3 Mb), which matched those identified in her oral epithelial sample, indicating they were non-pathogenic hereditary UPDs. Additionally, a 67 kb loss region on 8q24.13 was detected in the bone marrow sample but not in the oral epithelial sample, indicating it is an acquired lesion (Fig. 1). Gene exploration revealed that this region contains the *TRIB1* gene (Table 1). At the beginning of the disease, supportive treatments such as blood transfusion were mainly provided. As the condition progressed, we administered Azacytidine and low-dose cytarabine (Cytarabine). During the subsequent treatment observation, no gene mutations (mentioned above) or other chromosomal variations were found. Due to the patient's financial situation and personal requirements, further examinations such as next-generation sequencing (NGS) and the treatment option of hematopoietic stem cell transplantation (HSCT) were not considered. After three years’ follow-up, the disease progressed to MDS with increased blasts (MDS-IB2) from MDS-LB.

**Table 1:** Size and locations of the chromosome aberrations and candidate genes involved.

Chromosome	Lesion type	Size/Kb	Chromosome band	Location (Bone marrow cell, genome assembly: GRCh37.p13)	Location (Oral epithelial cell, genome assembly: GRCh37.p13)	Candidate genes involved
3	UPD	3335	p21.31-p21.2	48,712,421–52,047,421	48,712,421–52,047,421	*GPX1, KLHDC8B, TREX1, ADPRTL3*
8	Loss	67	q24.13	126,401,773–126,469,204	/	*TRIB1*
X	UPD	5526	p11.23–11.22	47,804,618–53,330,548	47,804,618–53,787,878	*ABCB7 GATA1, WAS*
X	UPD	5191	q11.1–12	61,932,503–67,123,772	61,932,503–67,866,291	
X	UPD	6344	q13.1–21.1	71,551,321–77,895,423	71,551,321–77,910,651	

## 3 Discussion

Cytogenetic analysis plays a pivotal role in unraveling MDS pathogenesis. The use of SNP-array has assisted adding new information to molecular mechanism, that is, the discovery of important gene mutations of MDS, for example, mutations of proto-oncogene *CBL, TET2* and *JARID2* [[3], [4], [5]]. These findings have greatly deepened our understanding of the molecular mechanism of MDS. Of the aberrations detected by SNP-array, copy number variations including gains and losses were frequently reported by a series of studies as they contributed to the genetic expression changes through dose effects. Although SNP-array karyotyping enhances aberration detection, distinguishing pathogenic mutations from non-pathogenic hereditary variations in bone marrow samples remains a critical challenge. Addressing this issue, our study used the patient's own oral epithelial DNA as a control to distinguish between somatic and hereditary variations. Notably, while four UPDs detected in marrow were confirmed as germline variations through tissue concordance analysis, the 8q24.13 microdeletion (67 kb) emerged exclusively in hematopoietic cells. This approach not only emphasizes the importance of using patient-specific controls in interpreting SNP-array results but also provides a practical method for identifying acquired pathogenic variants. Our findings advance current methodology by demonstrating how systematic comparison circumvents false-positive interpretations, thereby refining mutation prioritization in MDS diagnostics.

Emerging evidence delineates *TRIB1* as a molecular rheostat in myeloid malignancies, exhibiting context-dependent roles as both proto-oncogene (via RAS/MAPK-C/EBPα activation [[6], [7], [8]]) and tumor suppressor (through HDAC1-mediated-p53-deacetylation [9]). While murine models implicate *TRIB1* overexpression in leukemogenesis [7], its clinical relevance in human oncology disorders remains mechanistically unresolved. In some studies, *TRIB1* is identified as a transforming gene that accelerates leukaemogenesis, suggesting an oncogenic role [10]. However, in other settings, such as breast cancer, the role of *TRIB1* is more nuanced, with both overexpression and knockout promoting tumor growth [11], making its pathogenic mechanism more complex. In our study, we report the first documented case of somatic *TRIB1* copy number loss (8q24.13 deletion) associated with MDS. This aberration, undetectable by metaphase karyotyping but identified through SNP-array, more likely suggests a haploinsufficiency-driven pathogenesis permitting survival of dysplastic clones, which aligns with recent insights into tumor suppressor haploinsufficiency in myeloid neoplasms [12]. Given this is a single case report, the findings require validation in larger cohorts to assess the frequency and clinical relevance of *TRIB1* deletions in MDS. Additionally, functional studies are needed to confirm the mechanistic link between *TRIB1* haploinsufficiency and disease progression. Nevertheless, what we need to be aware of is *TRIB1*’s role in macrophage differentiation, particularly M2-like macrophages, as detailed in previous study [13], which suggests *TRIB1* may influence MDS’s immune microenvironment, offering a new research angle beyond its direct genetic impact, potentially affecting therapeutic strategies.

In conclusion, we identified the first somatic *TRIB1* gene copy number loss (8q24.13) in an MDS case, suggesting that its haploinsufficiency may contribute to clonal progression. By integrating SNP-array with patient-matched oral epithelial controls, somatic aberrations were distinguished from hereditary ones, enhancing diagnostic accuracy. Clinically, our study underscores the utility of advanced molecular profiling and patient-specific controls in refining diagnostics, aligning with evolving guidelines for myeloid malignancy management and highlights the need for further studies to clarify *TRIB1*'s role in MDS pathogenesis.

## Ethical approval and consent

This study adheres to the Declaration of Helsinki and was approved by the Ethics Committee of Qingdao Public Health Clinical Center. Written informed consent was obtained from the patient for publication of this case report and any accompanying images.

## CRediT authorship contribution statement

**Kun Chi:** Writing – original draft, Software, Data curation. **Lili Song:** Writing – review & editing, Investigation, Conceptualization.

## Declaration of competing interest

The authors declare no conflicts of interest.

## Data Availability

Data presented in this study are available from the corresponding author upon reasonable request.
